# Lp25 membrane protein from pathogenic *Leptospira* spp. is associated with rhabdomyolysis and oliguric acute kidney injury in a guinea pig model of leptospirosis

**DOI:** 10.1371/journal.pntd.0005615

**Published:** 2017-05-15

**Authors:** Patrícia A. E. Abreu, Antonio C. Seguro, Daniele Canale, Ana Maria G. da Silva, Larissa do R. B. Matos, Tatiane B. Gotti, Denize Monaris, Denise A. de Jesus, Sílvio A. Vasconcellos, Thales de Brito, Antonio J. B. Magaldi

**Affiliations:** 1 Laboratory of Bacteriology, Butantan Institute, São Paulo, Brazil; 2 Laboratory of Medical Investigation (LIM12), Clinical Hospital, Nephrology Department, São Paulo, Brazil; 3 Institute of Tropical Medicine, São Paulo University Medical School, São Paulo, Brazil; 4 Laboratory of Bacterial Zoonosis, School of Veterinary Medicine and Animal Science, University of São Paulo, São Paulo, Brazil; University of Minnesota, UNITED STATES

## Abstract

Acute kidney injury (AKI) from leptospirosis is frequently nonoliguric with hypo- or normokalemia. Higher serum potassium levels are observed in non-survivor patients and may have been caused by more severe AKI, metabolic disarrangement, or rhabdomyolysis. An association between the creatine phosphokinase (CPK) level and maximum serum creatinine level has been observed in these patients, which suggests that rhabdomyolysis contributes to severe AKI and hyperkalemia. LipL32 and Lp25 are conserved proteins in pathogenic strains of *Leptospira* spp., but these proteins have no known function. This study evaluated the effect of these proteins on renal function in guinea pigs. Lp25 is an outer membrane protein that appears responsible for the development of oliguric AKI associated with hyperkalemia induced by rhabdomyolysis (e.g., elevated CPK, uric acid and serum phosphate). This study is the first characterization of a leptospiral outer membrane protein that is associated with severe manifestations of leptospirosis. Therapeutic methods to attenuate this protein and inhibit rhabdomyolysis-induced AKI could protect animals and patients from severe forms of this disease and decrease mortality.

## Author summary

Rhabdomyolysis is a syndrome that results from the disruption of skeletal muscle integrity, leading to a massive release of the intracellular contents into the blood stream, including myoglobin, creatine phosphokinase, aspartate transaminase, lactate dehydrogenase, aldolase, and electrolytes. Complications of rhabdomyolysis include acute kidney injury (AKI), hyperkalemia, hyperphosphatemia, and hypovolemia, which may result in death without early treatment. The most frequent causes of this syndrome are trauma, excessive muscle activity, drugs, toxins, electrolyte imbalance, muscle ischemia, metabolic disorders, and infectious diseases. Among leptospirosis cases, the AKI induced by rhabdomyolysis has been described almost exclusively in patients with severe form of leptospirosis. However, the role of rhabdomyolysis in the pathogenesis of AKI due to leptospiral infection is not understood.

## Introduction

Leptospirosis is an emerging zoonosis that is caused by pathogenic spirochetes of the genus *Leptospira*. Approximately 1.03 million cases of the disease occur in humans worldwide, with approximately 60,000 deaths annually [1]. Many species of wild and domestic animals are leptospirosis reservoir hosts and eliminate leptospires to the environment via urinary shedding. Infection may result from direct contact with carrier animals or indirect contact with contaminated soil and water [2,3].

Human leptospirosis ranges from an asymptomatic or self-limited febrile illness (80 to 90% of cases) to a life-threatening illness (5 to 10% of cases). The life-threatening manifestation is characterized by Weil´s syndrome (renal failure, hemorrhage and jaundice) or severe pulmonary hemorrhagic syndrome [2–5].

Leptospirosis-induced acute kidney injury (AKI) is typically nonoliguric at the beginning of renal failure evolution or during mild forms with high frequency of hypokalemia [4]. In a prior study, higher serum potassium levels were observed in patients with more severe renal dysfunction concomitant with rhabdomyolysis. In addition, an association between creatine phosphokinase levels (CPK) (a marker of muscle injury) and maximum serum creatinine levels has been reported. This suggests that rhabdomyolysis is associated with severe AKI in leptospirosis [6].

Various bacterial, viral, fungal and protozoal infections lead to rhabdomyolysis [7–10], but the mechanism of muscle damage has not been established for many infections, including leptospirosis. Muscle injury may result from a direct pathogen invasion of skeletal muscle, tissue hypoxia, high lysosomal enzymatic activity or the release of toxins [11,12]. The identification of proteins that act as toxins in the host during leptospiral infection is essential to understanding the pathophysiological mechanisms of rhabdomyolysis and the mechanisms that contribute to severity of AKI.

The subsurface lipoprotein LipL32 is present in pathogenic leptospires, and it is the most abundantly expressed protein (40,000 copies per cell) [13,14]. However, the role of this protein in the pathogenesis of leptospirosis remains unknown [15]. Lp25 is a putative outer membrane lipoprotein of pathogenic *Leptospira* spp., but its function is not known. No sequences similar to this protein were identified in saprophytic *Leptospira* spp. [16–18].

The present study investigated whether the LipL32 and Lp25 proteins expressed by pathogenic *Leptospira* were associated with rhabdomyolysis and oliguric AKI in guinea pigs. To our knowledge, this study is the first characterization of a leptospiral protein associated with renal and muscular manifestations of leptospirosis.

## Methods

### *Leptospira* strains and culture

*Leptospira biflexa* serovar Patoc strain Patoc I, *Leptospira noguchii* serovar Panama strain CZ214K, *Leptospira borgpetersenii* serovar Javanica strain Veldrat Batavia 46, *Leptospira borgpetersenii* serovar Tarassovi strain 17, *Leptospira kirschneri* serovar Cynopteri strain 3522C, *Leptospira interrogans* serovar Hardjo strain Hardjoprajitno, *Leptospira interrogans* serovar Pomona strain 13A, and *Leptospira interrogans* serovar Copenhageni strain Fiocruz L1-130 were obtained from the Laboratory of Bacterial Zoonosis, School of Veterinary Medicine and Animal Science, University of São Paulo, Brazil. Leptospira strains were cultured at 29°C under aerobic conditions in liquid EMJH medium (Difco, Thermo Fisher Scientific, Boston, MA, USA.) with 10% rabbit serum, enriched with L-asparagine (0.015%), sodium pyruvate (0.001% [wt/vol]), calcium chloride (0.001% [wt/vol]), magnesium chloride (0.001% [wt/vol]), peptone (0.03% [wt/vol]), and meat extract (0.02% [wt/vol]) [17].

### Selection of proteins

LipL32 and Lp25 proteins were chosen for this study because no research has been performed to investigate their effects on renal function experimentally in animals. Lp25 was identified by bioinformatics analyses using the *L*. *interrogans* serovar Copenhageni strain Fiocruz L1-130 genome sequence previously described in studies published by our group [17]. The selection was based on the prediction of protein localization in the outer membrane. We gave priority to Lp25 because its function is not known. Leptospiral immunoglobulin-like protein A (LigA) [19] and LpL31 [20] were used as controls in the immunoblot analysis. LigA is a known outer membrane protein, and LipL31 is an inner membrane-associated protein [19, 20].

### Purification of recombinant proteins

Open reading frames LIC10009 (encoding a protein designated Lp25, for leptospiral protein 25, based on its molecular mass) [21] and LIC11352 (LipL32) were cloned into pAE [17,22] and pDEST-17 (Invitrogen, Carlsbad, CA, USA -or- Paisley, Scotland, UK.) vectors, respectively, as previously described [23]. The coding sequence of the carboxy-terminal portion of LigA (LigAC), corresponding to nucleotides 1891–3675 (LIC10465), was cloned into a pAE vector as previously described [21]. The coding sequence of the LipL31 (LIC11456) was amplified using PCR from genomic DNA of *L*. *interrogans* serovar Copenhageni strain Fiocruz L1-130 using the following primers: F: CTCGAGGGAGATAATTCCG and R: CTGCAGTTACTGCCCAGTAG. Sequences were digested using *Xho*I and *Hind*III restriction enzymes, and fragments were cloned into the pAE vector [22]. Competent cells of the *E*.*coli* BL21(DE3) strain were transformed with pAE-Lp25, pAE-LigAC, pDEST-LipL32, and pAE-LipL31 constructs and cultivated until the optical density at 600 nm reached 0.6. The expression of recombinant proteins was induced with 1 mM isopropyl-1-thio-β-D-galactopyranoside (IPTG) at 37°C for 3 h. The His-tagged Lp25, LipL32, LigAC, and LipL31 proteins were purified using metal affinity chromatography, as previously described [21].

### Production of polyclonal antisera against recombinant proteins

A New Zealand White rabbit was immunized for each protein via subcutaneous injection of 2 mg of purified recombinant protein absorbed in aluminum hydroxide as an adjuvant. The rabbits were immunized more two times with the same antigen preparation with fifteen day of interval. The rabbits were bled two weeks after last immunization. The IgG fraction from sera was precipitated using caprylic acid, as previously described [24].

### Isolation of leptospiral outer membrane proteins (OMPs)

OMPs from *L*. *interrogans* serovar Copenhageni strain Fiocruz L1-130 were extracted with Triton X-114 according to a previously described method [25]. Three distinct fractions were recovered: (P) the detergent-insoluble pellet that corresponds to inner membranes, cytoplasmic components, and non-lysed cells; (A) the aqueous phase that contains periplasmic content, and, (D) the Triton X-114 detergent phase with outer membranes. The whole cell lysate (W) and the obtained fractions were analyzed using immunoblots with LipL32, Lp25, LigA, and LipL31 antisera.

### Proteinase K accessibility assay

*L*. *interrogans* serovar Copenhageni strain Fiocruz L1-130 (5×10^8^ cells/ml) were harvested via centrifugation at 2000 × *g* for 7 min, gently washed with PBS containing 5 mM MgCl_2_, and collected via centrifugation at 2000 × *g* for 10 min. Washed spirochetes were gently resuspended in PBS-5mM MgCl_2_, and the evaluation of surface protein localization on intact leptospires was performed by treatment of proteinase K (PK—Sigma-Aldrich, St. Louis, MO, USA) as previously described [13]. Immunoblot analyses were performed using antibodies against LipL32 and Lp25.

### Immunoblotting

Proteins were transferred to a nitrocellulose membrane and probed with LipL32, Lp25, LigA, or LipL31 rabbit polyclonal antisera (diluted 1:500). The membrane was incubated with a secondary peroxidase-conjugated anti-rabbit antibody at a 1:5,000 dilution. The positive signals were detected using enhanced chemiluminescence (Thermo Fisher Scientific, Boston, MA, USA).

### Renal function

The guinea pigs were housed one per metabolic cages with food and drinking water freely available. The animals were acclimated to the housing conditions for 1 day prior to experimental procedures. Three groups of animals were studied: (1) control, n = 10 (1 ml of PBS); (2) LipL32, n = 13 (1 ml of PBS plus 400 μg of LipL32) and (3) Lp25, n = 14 (1 ml of PBS plus 400 μg of Lp25). The amount of protein used per dose (400 μg) was the greatest amount maintained in solution without precipitation. The solutions were injected intraperitoneally for 4 days. The animals were placed in metabolic cages for 12 h for urine collection on day 5. The volume of each 12-h urine sample was measured gravimetrically (UV ml/12 h). Urine samples were centrifuged in aliquots to remove suspended material, and urinary creatinine and sodium in the supernatants were measured. After urine collection all animals were anesthetized with a dose of sodium pentobarbital, and whole blood was collected by cardiac puncture. The animals were then killed with an overdose of anesthesia. Serum potassium and sodium were measured using flame photometry. The enzymatic colorimetric method (Labtest, Lagoa Santa, Brazil) was used to measure urinary and serum levels of creatinine, CPK and uric acid. The molybdate method was used to measure serum phosphate. The creatinine clearance (CrCl) was used to estimate the glomerular filtration rate (GFR) by formula: CrCl = U_cr_ (mg/dL) x UV (ml/min) / P_cr_ (mg/dL), corrected by 100 g of body weight (ml/min/100 g body weight) [26]. Fractional excretion of sodium was calculated using the formula: FE_Na_ = U_Na_ x P_Cr_ / P_Na_ x U_Cr_ x 100%. AKI was defined as a decrease in the GFR of more than 50% from the mean value obtained in the control group (PBS), and oliguria was defined as a urinary output of less than 50% of mean value of control group (PBS). Rhabdomyolysis was defined as an elevation in serum CPK of at least 3 times the mean value obtained in the control group. The occurrence of hyperkalemia, hyperphosphatemia, and hyperuricemia was defined as an increase of phosphate, potassium, and acid uric levels of more than the mean value of control group (PBS).

### Immunohistochemistry and histology analyses

Muscle fragments from legs and paravertebral regions were collected at the time of euthanasia, routinely fixed in 10% buffered formalin (pH 7.2), embedded in paraffin and sectioned at 3 μm. Fragments from kidney were also collected and submitted to routine histological procedures. Sections were analyzed using EnVision (Dako, Glostrup, Denmark)-based immunohistochemistry methods, as previously described (5). The antigen retrieval step was performed by pressure cooking in 10 mM sodium citrate pH 6. Following the overnight incubation with primary rabbit polyclonal antisera (diluted 1:3,000–1:4,000) at 4°C and with secondary antibody (Envision peroxidase Dako K4002) for 30 min at room temperature. The presence of nonspecific staining was assessed using preimmune sera. Tissue sections for morphological analyses were stained with hematoxylin and eosin (H&E) and Gomori trichrome stain in selected sections. Muscular lesions were graded on a scale from 0 to 2: 0 as normal (without lesions), 1 as mild (chiefly the presence of individual hyaline contraction change and focal inflammatory interstitial reactivity), and 2 as severe (presence of necrosis, multiple lesions of individual myocytes and interstitial inflammatory infiltrated). Kidney sections were also fixed in 10% buffered formalin and stained with H&E for morphological analyses. Images were captured on an Axiophot Zeiss Axio microscope and analyzed using AxionVision 4.6 software.

### Ethics statement

The Ethic Committee on Animal Use of the Butantan Institute (CEUAIB), São Paulo, Brazil, previously approved the experimental protocols under the license numbers 55708 for the rabbit procedure and 99112 for guinea pig procedure. All animal procedures were conducted following the rules issued by the Brazilian National Council for Control of Animal Experimentation (CONCEA).

### Statistical analysis

All quantitative data are expressed as the means ± SEM. Differences between the means of multiple parameters were analyzed using ANOVA followed by Student-Newman-Keuls test. Histological scores were compared using Student’s t-test. Values of p < 0.05 were considered statistically significant. All analyses were performed using GraphPad Prism 5 (Graphpad, La Jolla, CA).

## Results

### Lp25 is associated with rhabdomyolysis and oliguric acute kidney injury

**Fig 1:**
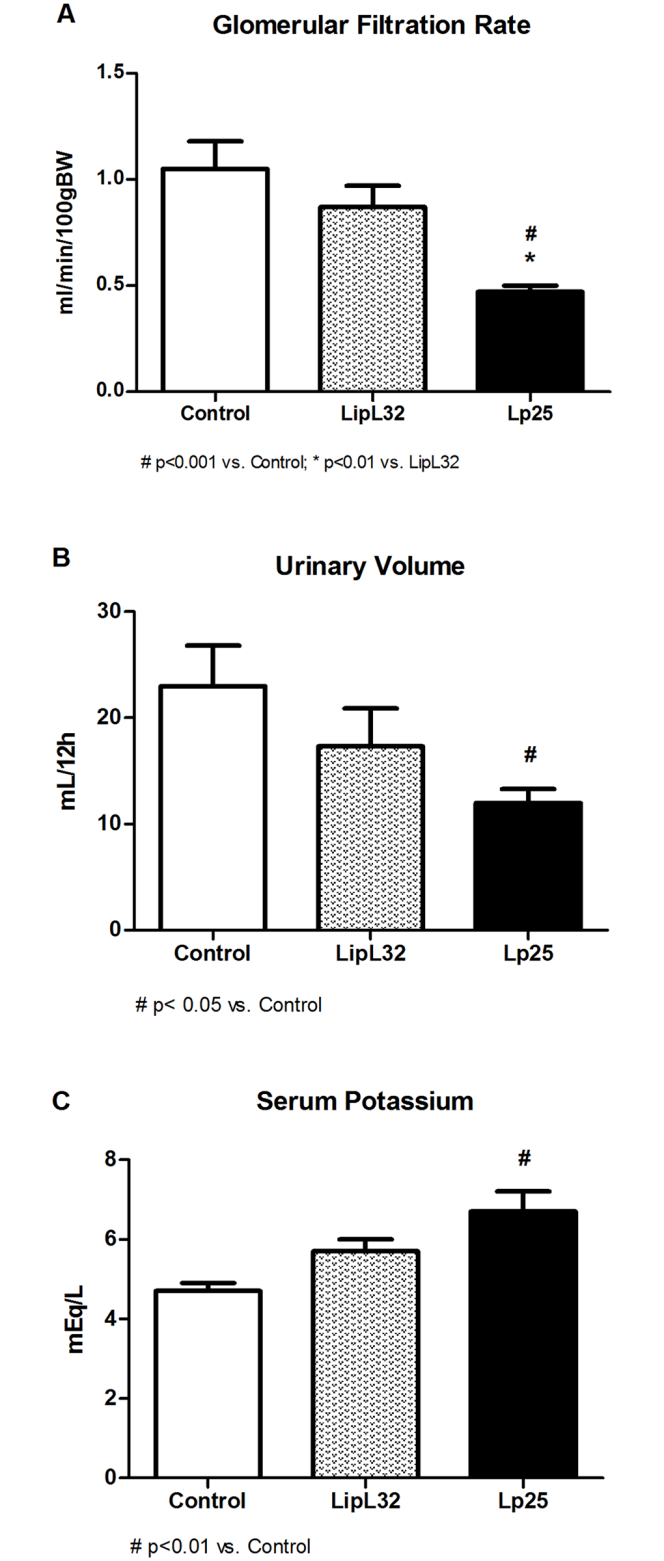
Biochemical parameters of renal function in guinea pigs injected intraperitoneally with recombinant proteins or PBS (control). (A) GFR evaluated using creatinine clearance. (B) Urinary volume values. (C) Serum potassium values. Data are means ± SEM.

A total of 37 guinea pigs were assigned into one of three treatment groups: control (n = 10), LipL32 (n = 13) or Lp25 (n = 14). Initial body weights were similar between the 3 groups: 194±4 g (control), 195±4 g (LipL32) and 191±3 g (Lp25). Weight gain was lower in the LipL32 group (18±3 g, p<0,05) and Lp25 group (13±3 g, p<0,01) than the control group (28±2 g) on day 5. Fig 1 shows that the GFR, evaluated as creatinine clearance (CrCl), was significantly lower in the Lp25 group (0.47±0.03 mL/min/100 gBW) than the control group (1.05±0.13 mL/min/100 gBW) and LipL32 (0.87±0.10 mL/min/100 gBW). The urinary volume was lower in the Lp25 group (12.0±1.3 UV mL/12h) than the control group (23.0±3.8 UV mL/12h) and the LipL32 group (17.3±3.6 UV mL/12h). Notably, the serum potassium level in the Lp25 group (6.7±0.5 mEq/L) was elevated, compared to the control group (4.7±0.2 mEq/L) and the LipL32 group (5.7±0.3 mEq/L). The fractional excretion of sodium was similar in the three groups (control 0.82±0.18%; LipL32 0.60±0.09%; Lp25 0.83± 0.15%).

**Fig 2:**
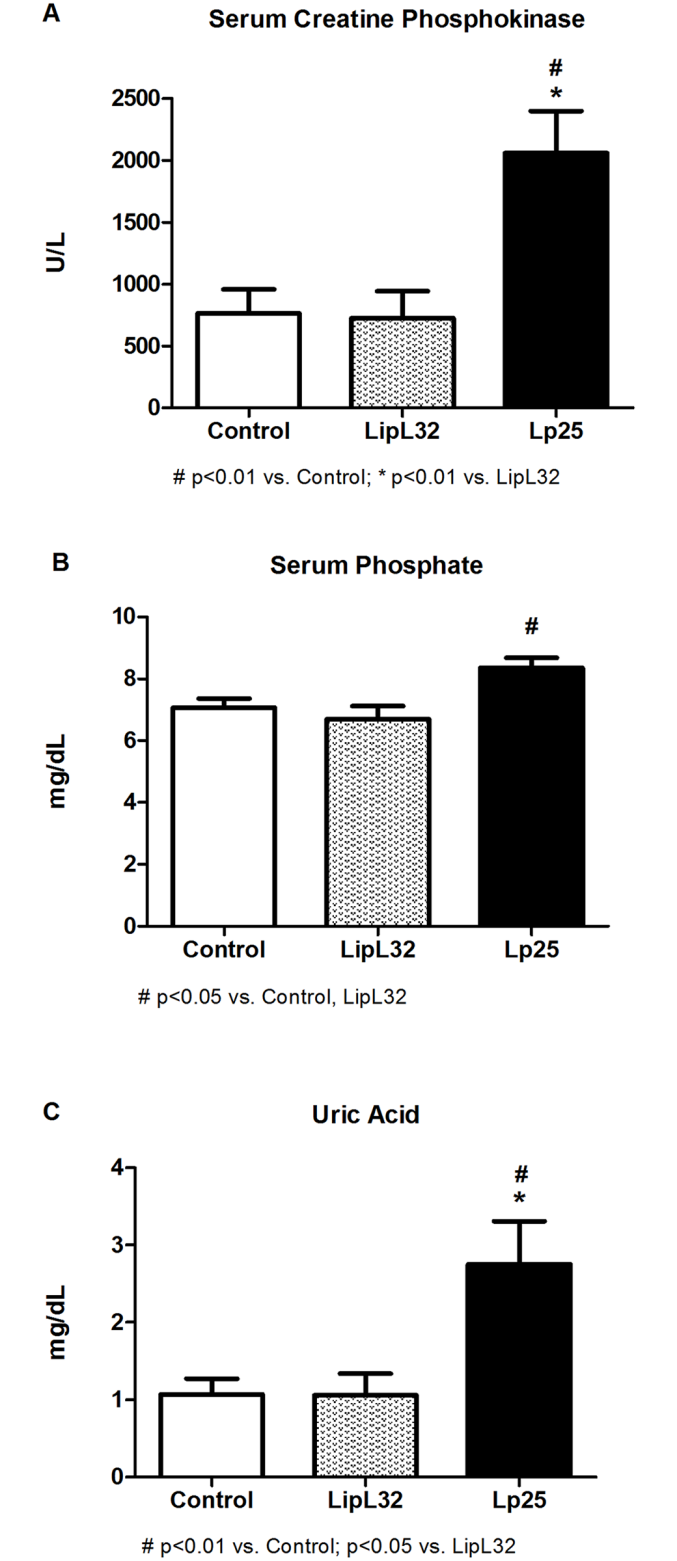
Biochemical parameters of rhabdomyolysis in guinea pigs injected intraperitoneally with recombinant proteins or PBS (control). (A) Serum creatine phosphokinase values. (B) Serum phosphate values. (C) Uric acid values. Data are means ± SEM.

The Lp25 group had significantly higher levels of serum CPK, phosphate and uric acid (2060±338 CPK U/L; 8.36±0.32 P mg/dL and 2.75±0.56 acid uric mg/dL) (Fig 2). These parameters in the LipL32 group (726±216 CPK U/L; 6.70±0.41 P mg/dL and 1.06±0.28 acid uric mg/dL) were similar to the control group (763±197 CPK U/L; 7.06±0.30 P mg/dL and 1.07±0.20 acid uric mg/dL).

AKI was observed in all animals of Lp25 group (100%) and 7/14 (50%) of these also had oliguria. In the LipL32 group, 2/13 animals (15.38%) had AKI oliguric and 1/13 (7.69%) presented AKI nonoliguric. Hyperkalemia was seen in 13/14 (92.85%) guinea pigs in the Lp25 group vs 8/13 (61.53%) in the LpL32 group.

In the Lp25 group, rhabdomyolysis, hyperphosphatemia, and hyperuricemia were encountered in 12/14 (85.71%), 10/14 (71.42%) and 9/14 (64.28%) animals, respectively. Seven animals of this group (50%) showed the three outcomes concomitantly. In the LipL32 group, 2/13 (15.38%) had rhabdomyolysis, 3/13 (23.07%) had hyperphosphatemia and 2/13 (15.38%) had hyperuricemia.

### Lp25 induced muscular lesions that were more accentuated than LipL32

**Fig 3:**
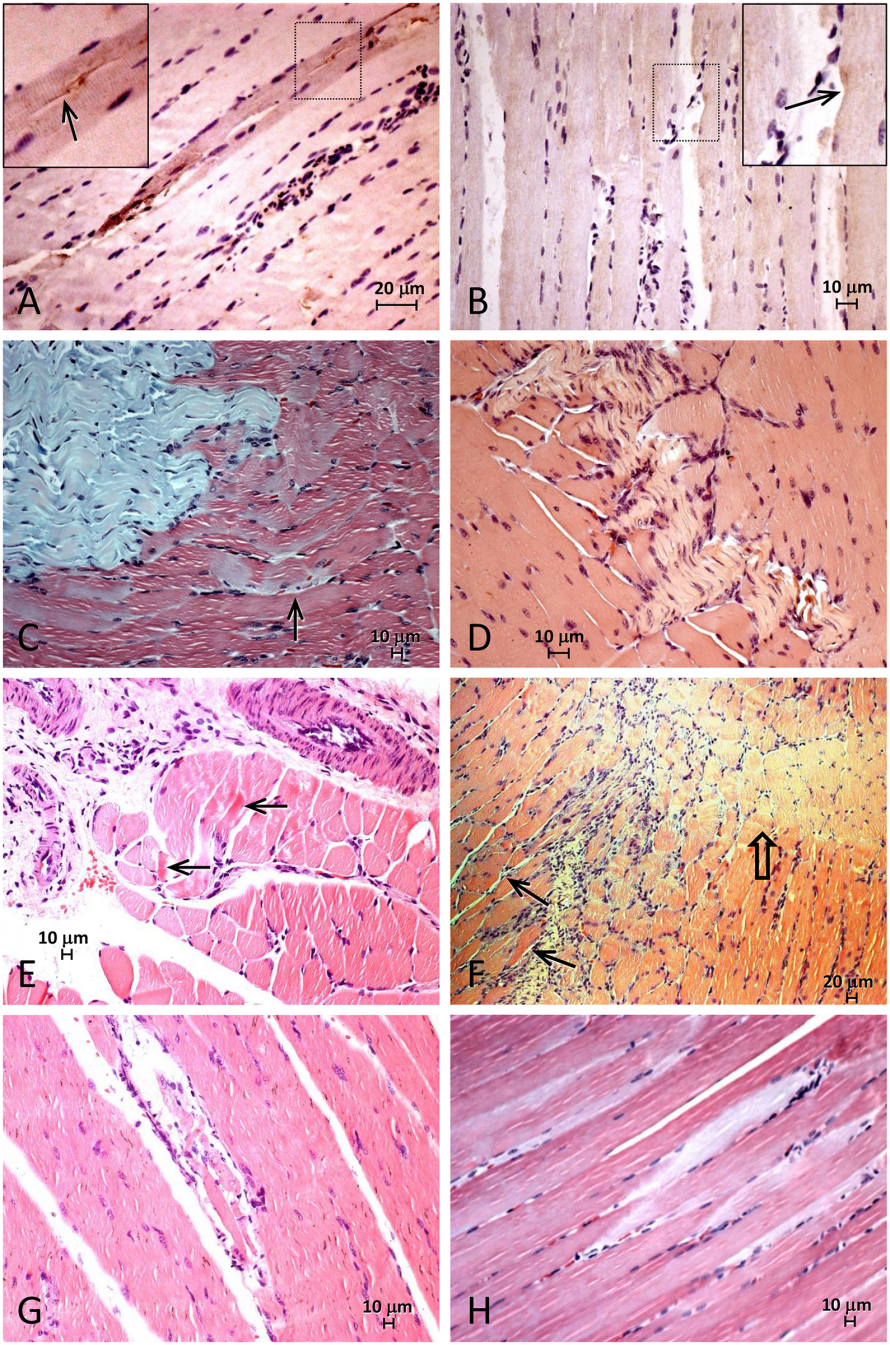
Immunohistochemistry and histology analyses of muscle fragments from guinea pigs injected intraperitoneally with Lp25 and LipL32 recombinant proteins. (A) Lp25 –Immunohistochemistry showing brownish granular cytoplasmatic antigen. Inset details antigen deposits over the sarcolemma (arrow). Discrete interstitial inflammation is also present. (B) LipL32 –Immunohistochemistry showing similar deposits of antigen in the cytoplasm of muscular cells and (see inset) on the sarcolemma (arrow). (C) Lp25—Gomori stain showing necrosis (stained in blue) and the presence of areas of “ghost cells” at the periphery of the muscle (arrow). (D) LipL32 –H&E stain showing areas of muscular necrosis. (E) Lp25 –H&E stain with focal areas of inflammatory infiltrate primarily composed of mononuclear cells. Arrows point to muscular cells undergoing hyaline contraction. (F) Lp25 –H&E stain showing interstitial inflammation primarily composed of mononuclear cells around vessels and muscle periphery. Arrow points to muscular cells undergoing hyaline contraction. Short arrow points to area of pale muscular cells without myofibrils. (G) Lp25 –H&E stain. Hyaline necrosis of muscular cells surrounded by mononuclear chronic inflammatory infiltrate (possible phagocytosis of the cellular remnants). (H) LipL32—Gomori stain. Muscle fibers showing empty spaces delineated by remnants of sarcolemma (“ghost cells”).

We performed immunohistological and morphological analyses to assess the effect of the LipL32 and Lp25 proteins on muscle tissues. The anti-rabbit Lp25 antibodies labeled isolated or small groups of muscular fibers with granular, faintly brownish antigen deposits that may partially delineate the sarcolemma and spread to the cytoplasm below (Fig 3A). The inflammatory infiltrate was generally discrete and primarily composed of small groups of monocytes that were present as focal isolated interstitial groups or small groups of isolated muscular fibers, frequently with antigenic linear deposits that partially circumscribed muscular fibers or inside their cytoplasm. Cytoplasmic antigenic granules were also detected occasionally in mononuclear phagocytic cells when the inflammatory infiltrate is more conspicuous. Immunohistochemistry with the anti-rabbit LipL32 antibodies was also positive on the sarcolemma and in the cytoplasm of isolated muscular fibers. Scarce mononuclear inflammatory interstitial reactions near the damaged muscle were also present (Fig 3B). Histological analyses of the muscle fragments revealed essentially similar muscular lesions were present in animals inoculated with both proteins, but with different degrees of severity (Fig 3C–3H). No wide range of muscular fiber sizes (small or large groups of atrophic or hypertrophic fibers) were observed in either protein group. Internal sarcolemmal nuclei were not detected in isolated muscle fibers. However, isolated nonspecific muscular lesions were present and ranged from hyaline contraction cytoplasmatic changes (Fig 3E and 3F) to small vacuoles, which may progress to hypercontracted isolated fibers to necrosis prior to phagocytosis (Fig 3G) or to intermediate damage, which was characterized by staining changes in myofibrils (Fig 3F), including the appearance of pale necrotic cells in H&E and Gomori trichrome stains (“ghost cells “) (Fig 3F and 3H). Irregular areas of muscle necrosis were the end result of these muscular disturbances (Fig 3C and 3D). Mild inflammatory infiltrate composed of macrophages were also observed around necrotic areas. Fragments from the LipL32 group revealed essentially similar findings to the Lp25 group, but with less frequent focal muscular damage and areas of necrosis (Fig 3D).

**Fig 4:**
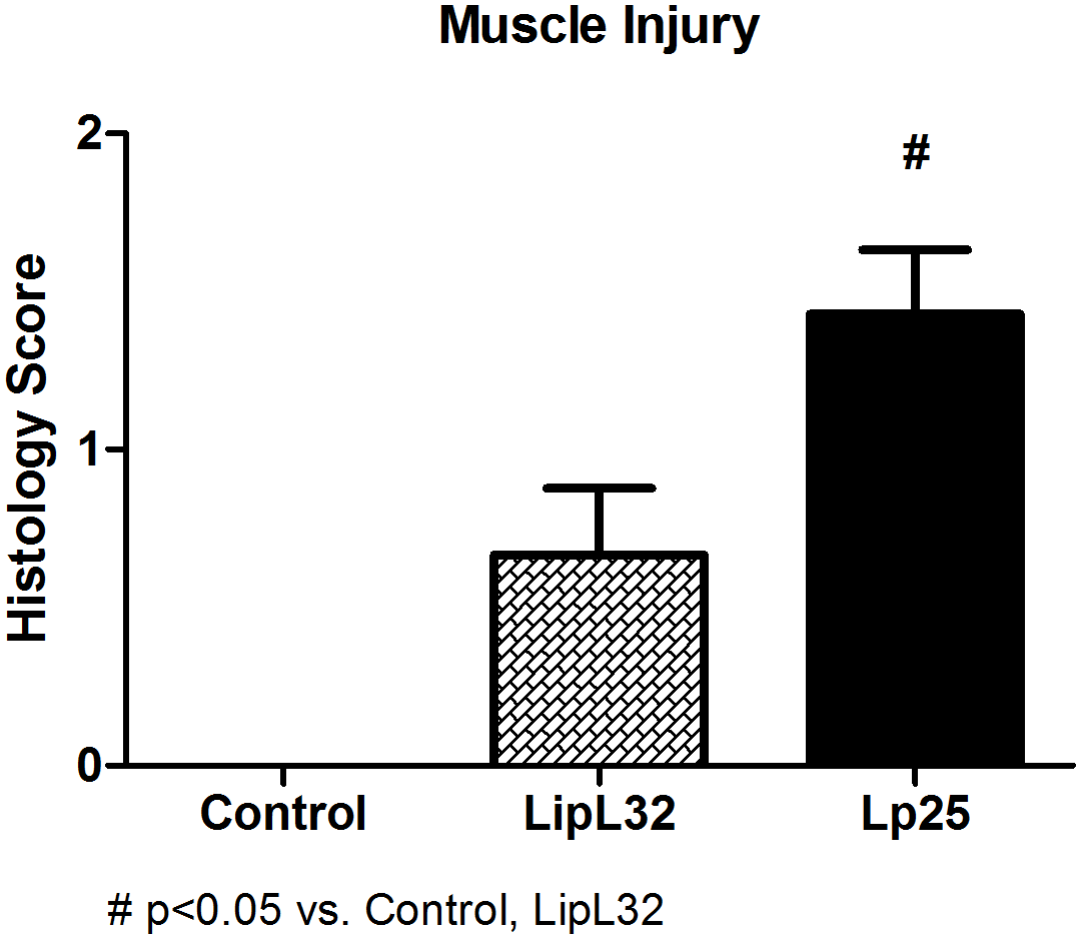
Muscle histology score. Legs and paravertebral regions fragments were collected from guinea pigs injected intraperitoneally with recombinant proteins or PBS (control) and stained with H&E and Gomori´s trichrome. The muscular lesions were graded as described in the Material and Methods. Data are means ± SEM.

Muscular lesions severity scores were significantly lower (p<0.05) in the LipL32 group (0.66 ± 0.21) than the Lp25 group (1.43 ± 0.20), and no muscular lesions were observed in the control group. The difference between LipL32 and Lp25-inoculated animals was statistically significant (p<0.05, LipL32 vs. Lp25) (Fig 4). Specifically, 53.85% of animals treated with LipL32 shown no muscular lesions, whereas the lesions of severity stage 1 and 2 were observed in 30.77% and 15.38%, respectively, of guinea pigs inoculated with this protein. Otherwise, all Lp25-inoculated animals presented muscular lesions, of these 42.86% had mild manifestations (stage 1) and 57.14% had severe signs (stage 2). Histological examination of the kidneys revealed no lesions (S1 Fig).

### Lp25 protein is conserved in pathogenic strains of *Leptospira* spp.

**Fig 5:**
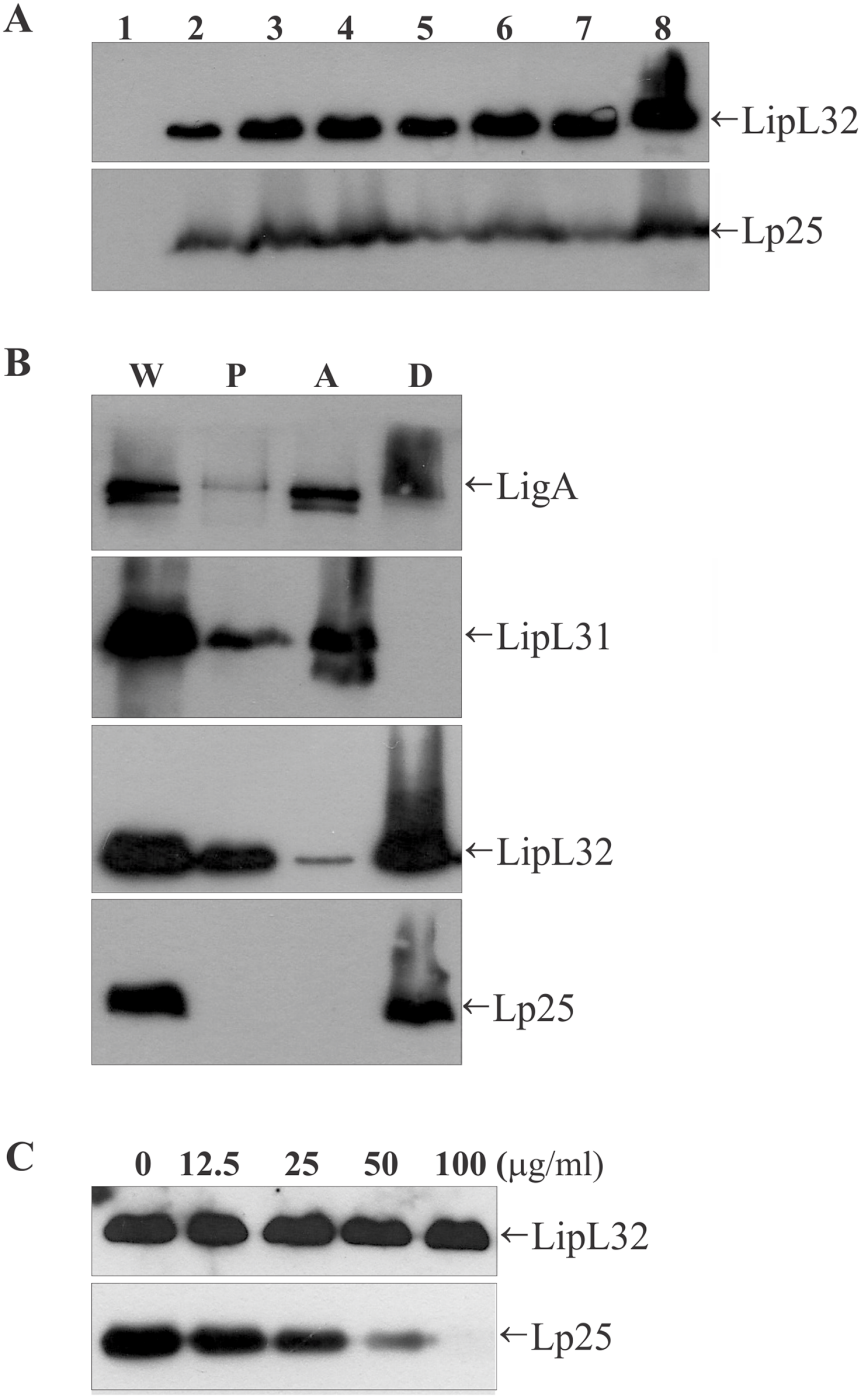
Expression in *Leptospira* species and surface localization of the Lp25 protein. (A) Whole-cell lysates were analyzed by immunoblotting using anti-LipL32 and anti-Lp25 antisera. Saprophytic *Leptospira biflexa* serovar Patoc strain Patoc I (Lane 1) and pathogenic species: *L*. *noguchii* serovar Panama strain CZ 214K (Lane 2), *L*. *borgpetersenii* serovar Javanica strain Veldrat Batavia 46 (Lane 3), *L*. *borgpetersenii* serovar Tarassovi strain 17 (Lane 4), *L*. *kirschneri* serovar Cynopteri strain 3522C (Lane 5), *L*. *interrogans* serovar Hardjo strain Hardjoprajitno (Lane 6), *L*. *interrogans* serovar Pomona strain 13A (Lane 7), and *L*. *interrogans* serovar Copenhageni strain L1-130 (Lane 8). (B) Triton X-114 fractions of *L*. *interrogans* serovar Copenhageni strain L1-130 were analyzed by immunoblotting with anti-Lp25 antiserum. As controls, membranes were probed with antisera against LigA (surface protein), LipL31 (cytoplasmatic protein) and LipL32 (subsurface protein). The fractions analyzed were whole lysate (W), Triton X-114-insoluble pellet (P), the aqueous phase (A), and the detergent phase (D). (C) Proteinase K accessibility assay. Whole intact leptospires were incubated with different concentrations of proteinase K and processed for immunoblot analyses using antibodies against Lp25 or LipL32.

Immunoblots of whole cell lysates revealed that the Lp25 protein was expressed by all strains of pathogenic leptospires tested, and it was not detected in the non-pathogenic strain (Fig 5A). Equal results were obtained in immunoblot assays using LipL32 antiserum as a positive control and demonstrated that the Lp25 protein, like the LipL32 [25], was conserved and only found in pathogenic species of *Leptospira*.

### Lp25 is an outer membrane protein

All control proteins were detected in whole cell extracts. LigA was completely solubilized by the detergent and fractionated into the aqueous and detergent phases. LipL31 was detected in the insoluble pellet fraction and aqueous phase, and it was completely absent from the detergent phase (Fig 5B). We performed an additional experiment using the LipL32 antiserum because a previous work reported that LipL32 was solubilized by Triton X-114 and mostly detected in the detergent fraction [25]. Fig 5B shows that the presence of LipL32 in the Triton X-114 fraction was confirmed [25]. These results demonstrated the correct functioning of the Triton X-114 fractionation method.

We also investigated the surface localization of Lp25 using proteolysis of intact cells of the *L*. *interrogans* serovar Copenhageni strain L1-130 using proteinase K. Fig 5C shows that Lp25 was susceptible to protease treatment in a dose-dependent manner, and the subsurface LipL32 was not susceptible, which suggests that Lp25 is exposed on the surface. These results are consistent with a previously published study that demonstrated that LipL32 was not exposed on the leptospiral surface, despite its localization in the outer membrane [13].

## Discussion

The renal manifestations of leptospirosis are variable and range from mild symptoms, such as low urinary protein excretion and sediment changes, to fatal AKI [4,27,28]. Severe cases of AKI are generally oliguric and hyperkalemic with a prolonged course and high mortality rate. Nonoliguric and normo- or hypokalemic AKI-forms are associated with a better prognosis [4, 27–29]. The renal pathophysiology that is consequent to leptospirosis infection is not clearly known despite advances in the knowledge of AKI epidemiology [4,5, 28–32]. Different factors may be involved, such as inflammatory processes, rhabdomyolysis, hemodynamic alterations, immune responses, and direct effects of leptospires and their products [30, 33, 31].

This study evaluated the effect of the LipL32 and Lp25 proteins on renal function in normal guinea pigs. We found that only Lp25 was associated with the development of oliguric AKI and rhabdomyolysis-induced hyperkalemia (elevated CPK, uric acid and serum phosphate).

Lp25 decreased the GFR compared LipL32 and control experiments (Fig 1). These results demonstrate, for the first time, that a specific protein from pathogenic *Leptospira* spp. plays an important role in the establishment of the AKI that is observed in Weil’s syndrome. In contrast, LipL32 protein did not produce a decrease in the GFR, despite a report that LipL32 induced interstitial nephritis-mediated gene expression in cultured mouse proximal tubule cells [34] and acute tubular injury in proximal pronephric ducts from zebrafish larvae kidneys [35]. These experiments were performed using *in vitro* preparations and another animal species. The decrease in GFR may not be directly dependent on the tubular damage because the FE_Na_ was not different between the three groups. Previous clinical studies also demonstrated that rhabdomyolysis-induced acute renal injuries did not modify sodium excretion [36–38].

Our results from cellular localization assays agree with previous studies that demonstrated that LipL32 was a subsurface protein that was not accessible on the leptospiral surface [13]. Notably, the role of LipL32 in Leptospira biology is not defined despite its abundant expression [14] in all pathogenic serovars. Murray and colleagues (2009) demonstrated that LipL32 was not required in acute (hamster) or chronic (rat) infection models for leptospirosis [39].

The results in Figs 1 and 2, such as the increase in potassium, creatine phosphokinase, uric acid and phosphate serum levels, are characteristics of the presence of rhabdomyolysis. The classification of the muscular lesions observed in histological preparations using a score (Fig 4) revealed that Lp25 induced the same type of muscular lesions as LipL32, but the Lp25 lesions were much more severe. Fig 3 shows areas of necrosis and moderate inflammatory infiltrate in the Lp25 group, and the LipL32 group exhibited small areas of necrosis and few inflammatory areas. Immunohistochemistry with anti-rabbit Lp25 and LipL32 antisera were positive for the two proteins and both proteins exhibited the same histological patterns. The deposition of the antigens was more intense in the Lp25 group. These results suggested that the LipL32 and Lp25 proteins reached the muscle tissue and induced lesions. However, lesions in the Lp25 group were more diffuse and apparently more accentuated than the LipL32 group.

We also demonstrated that Lp25 was a surface-exposed and conserved protein in pathogenic species of *Leptospira*. Previously published immunoblot studies using sera from leptospirosis patients and infected hamsters showed that Lp25 protein was expressed during the course of leptospiral infection [40,21]. This protein was recently included in the *Leptospira* endostatin-like (Len) family by the automatic NCBI prokaryotic genome re-annotation pipeline [41]. The members of the Len family bind plasminogen, laminin and human complement regulator factors [42–44, 18]. However, we previously demonstrated that Lp25 did not exhibit extracellular matrix-binding properties or play a role in immune evasion via interacting with the human complement regulator C4BP [17,45]. Comparative proteomic analyses of leptospira outer membrane proteins also demonstrated that the Lp25 protein, encoded by the LA0009 gene in *L*. *interrogans* serovar Lai, was up-regulated (1.3-fold) after an overnight upshift to 37°C [46]. These features are also compatible with one of the potential roles of the Lp25 protein, which is causing muscular damage that consequently is associated with oliguric AKI and hyperkalemia. These data demonstrated, for the first time, that Lp25 is associated with rhabdomyolysis, which is an important sign in leptospirosis and may underlie the muscular pain, which is a pathognomonic symptom of this disease.

## Supporting information

S1 Fig Histology analysis of kidney fragment from guinea pig inoculated with Lp25 protein. H&E stain showing cortical region with glomeruli, tubules and interstitium without pathological findings.
(TIF)
