# Supplementary figures and images for: Lp25 membrane protein from pathogenic Leptospira spp. is associated with rhabdomyolysis and oliguric acute kidney injury in a guinea pig model of leptospirosis
Source: PLoS Negl Trop Dis. 2017 May 15;11(5):e0005615. doi: 10.1371/journal.pntd.0005615 (PMC5444857; doi:10.1371/journal.pntd.0005615)

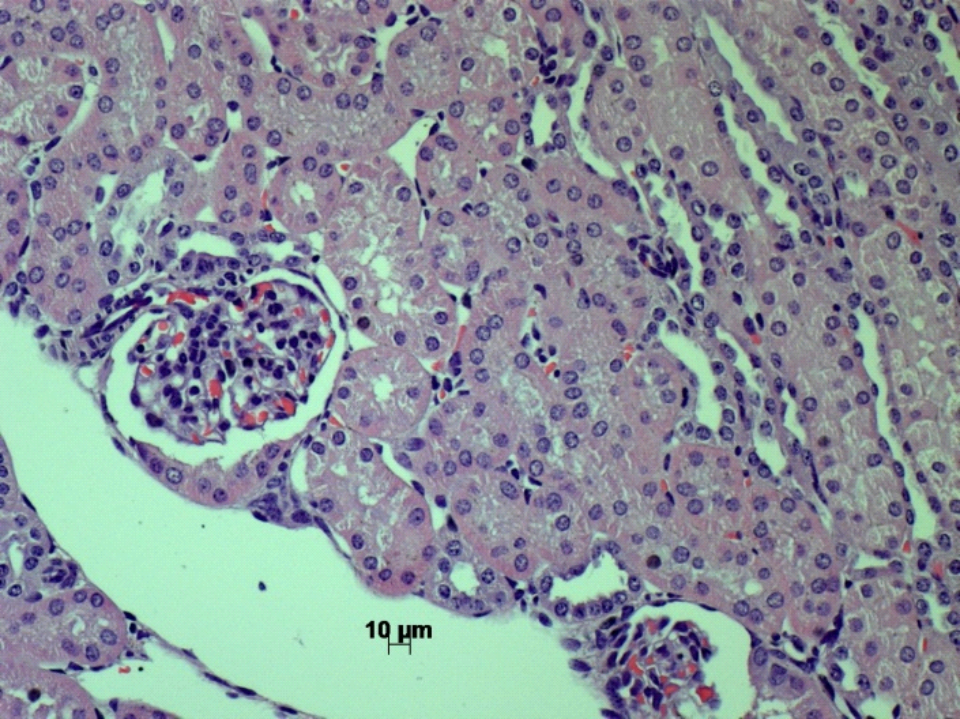

Supplement: S1 Fig — H&E stain showing cortical region with glomeruli, tubules and interstitium without pathological findings. (TIF) [file pntd.0005615.s001.tif]
